# Longitudinal soluble marker profiles reveal strong association between cytokine storms resulting from macrophage activation and disease severity in COVID-19 disease

**DOI:** 10.1038/s41598-024-63586-8

**Published:** 2024-06-05

**Authors:** Krista E. van Meijgaarden, Suzanne van Veen, Roula Tsonaka, Paula Ruibal, Anna H. E. Roukens, Sesmu M. Arbous, Judith Manniën, Suzanne C. Cannegieter, Tom H. M. Ottenhoff, Simone A. Joosten, BEAT-COVID group, Sesmu M. Arbous, Bernard M. van den Berg, Suzanne Cannegieter, Christa M. Cobbaert, Anne M. van der Does, Jacques J. M. van Dongen, Jeroen Eikenboom, Mariet C. W. Feltkamp, Annemieke Geluk, Jelle J. Goeman, Martin Giera, Thomas Hankemeier, Mirjam H. M. Heemskerk, Pieter S. Hiemstra, Cornelis H. Hokke, Jacqueline J. Janse, Simon P. Jochems, Marjolein Kikkert, Lieke Lamont, Tamás Pongrácz, Michael R. del Prado, Meta Roestenberg, Hermelijn H. Smits, Eric J. Snijder, Frank J. T. Staal, Leendert A. Trouw, Aswin Verhoeven, Leo G. Visser, Jutte J. C. de Vries, David J. van Westerloo, Jeanette Wigbers, Henk J. van der Wijk, Robin C. van Wissen, Manfred Wuhrer, Maria Yazdanbakhsh, Mihaela Zlei, COVID-19 LUMC group, Josine A. Oud, Meryem Baysan, Jeanette Wigbers, Lieke J. van Heurn, Susan B. ter Haar, Alexandra G. L. Toppenberg, Laura Heerdink, Annekee A. van IJlzinga Veenstra, Anna M. Eikenboom, Julia M. Wubbolts, Jonathan Uzorka, Willem Lijfering, Romy Meier, Ingeborg de Jonge, Sesmu M. Arbous, Mark G. J. de Boer, Anske G. van der Bom, Olaf M. Dekkers, Frits Rosendaal

**Affiliations:** 1 Department of Infectious Diseases, Leiden University Medical Center, Albinusdreef 2, 2333 ZA Leiden, The Netherlands; 2 Department of Biomedical Data Sciences, Leiden University Medical Center, Leiden, The Netherlands; 3 Department of Intensive Care Medicine, Leiden University Medical Center, Leiden, The Netherlands; 4 Department of Clinical Epidemiology, Leiden University Medical Center, Leiden, The Netherlands; 5 Department of Intensive Care, Leiden University Medical Center, Leiden, The Netherlands; 6 Department of Internal Medicine, Leiden University Medical Center, Leiden, The Netherlands; 7 Department of Clinical Chemistry, Leiden University Medical Center, Leiden, The Netherlands; 8 Department of Pulmonology, Leiden University Medical Center, Leiden, The Netherlands; 9 Department of Immunology, Leiden University Medical Center, Leiden, The Netherlands; 10 Department of Medical Microbiology, Leiden University Medical Center, Leiden, The Netherlands; 11 Center for Proteomics and Metabolomics, Leiden University Medical Center, Leiden, The Netherlands; 12 Division of Systems Biomedicine and Pharmacology, Leiden Academic Center for Drug Research, Leiden University, Leiden, The Netherlands; 13 Department of Hematology, Leiden University Medical Center, Leiden, The Netherlands; 14 Department of Parasitology, Leiden University Medical Center, Leiden, The Netherlands

**Keywords:** Immunology, Cytokines, Chemokines, Biomarkers, Diseases, Infectious diseases

## Abstract

SARS-CoV2 infection results in a range of disease severities, but the underlying differential pathogenesis is still not completely understood. At presentation it remains difficult to estimate and predict severity, in particular, identify individuals at greatest risk of progression towards the most severe disease-states. Here we used advanced models with circulating serum analytes as variables in combination with daily assessment of disease severity using the SCODA-score, not only at single time points but also during the course of disease, to correlate analyte levels and disease severity. We identified a remarkably strong pro-inflammatory cytokine/chemokine profile with high levels for sCD163, CCL20, HGF, CHintinase3like1 and Pentraxin3 in serum which correlated with COVID-19 disease severity and overall outcome. Although precise analyte levels differed, resulting biomarker profiles were highly similar at early and late disease stages, and even during convalescence similar biomarkers were elevated and further included CXCL3, CXCL6 and Osteopontin. Taken together, strong pro-inflammatory marker profiles were identified in patients with COVID-19 disease which correlated with overall outcome and disease severity.

## Introduction

Infection with SARS-CoV2 triggers the immune system, however, infection-outcome remains unpredictable and highly variable. Individuals infected with SARS-CoV2 can present in a wide range, from completely asymptomatic to severe disease requiring mechanical ventilation or even fatal outcome.

Infection of the airway epithelium with SARS-CoV2 results in rapid immune activation, with strongly increased pro-inflammatory cytokines and chemokines in the circulation of patients with severe COVID-19^1^. Profiling circulating markers in COVID-19 suspects at the emergency department identified 14 proteins associated with SARS-CoV2 infection^2^ and 12 markers associated with hospitalization (more severe disease), while five markers predicted fatal outcome^2^. The type of markers associated with the different outcomes suggests wide activation of the immune system with a strong pro-inflammatory character.

In other work, patients with severe COVID-19 disease had increased plasma levels of HGF (hepatocyte growth factor), AREG (amphiregulin), CKAP4 (cytoskeleton-associated protein 4), S100A12, NCF2 (neutrophil cytosolic factor-2) and ITGB6 (integrin beta-6), relative to individuals with moderate COVID-19 disease^3^. During convalescence (4 months after acute disease) most markers normalized to levels of healthy controls, except for HGF and KRT19 (cytokeratin-19)^3,4^. The combination HGF/CXCL13 was the best predictor for ICU-admission but also fatal outcome in 3 combined independent cohorts of COVID-19 patients^5^. HGF has a role in tissue repair, including the lungs and has anti-inflammatory properties which are considered to regulate excessive pro-inflammatory cytokines to limit tissue-damage^5^. In individuals presenting with pulmonary symptoms at the emergency department, circulating inflammatory markers were compared in individuals with COVID-19 to those with similar symptoms by other causes^4^. Patients with PCR-confirmed COVID-19 had higher levels of IFN-related markers and other viral response related molecules^4^. Disease severity was predicted at time of presentation in the hospital, and the strongest factors in the predictive signature were IL-6, IL-1RL1, Pentraxin 3, IL-1N (inhibitor of IL-1 receptor), KRT-19 and TRIAP1 (TP53 Regulated Inhibitor Of Apoptosis 1)^4^. This suggests that it is mostly the strength of the pro-inflammatory response at hospital admission that predicts disease severity over all. Since patients with COVID-19 disease had different plasma protein profiles compared to patients with sepsis and pneumonia, but a very similar clinical presentation, the resulting signatures were able to discriminate sepsis with pneumonia from COVID-19-induced respiratory distress^3,4^.

Early during the pandemic strong inflammation was recognized as hallmark of COVID-19 disease, and specific targeting strategies were sought and examined to minimize inflammation associated damage without affecting viral elimination^2,4,6^. IL-6 targeting successfully reduced mortality and disease duration in critically-ill patients^6–10^, accompanied by long-term beneficial effects^11^. Also the administration of anakinra (a selective IL-1 inhibitor) increased the chance of recovery, in most patients^6,12–15^. Furthermore, interventions targeting type-I, but not type-II interferons had beneficial outcomes^6,16^. Thus, specific modulation of pro-inflammatory pathways in the early stages of disease pathogenesis may control fulminant inflammation and resulting tissue damage, suggesting a critical, possibly causal, contribution of inflammation to disease.

The plethora of available data sets are diverse and difficult to compare as many studies have used rather global categories for severity. Severity was in some studies only defined as outcome measure, or as status at the moment of sample collection, i.e. mild, moderate or severe infection, based on the location of the patient (outpatient, hospital ward-admitted or ICU -admitted). However, better pathophysiological insights can be obtained using more careful definition and stratification of patients, as well as by longitudinal assessment of changes over different infection-phases. This not only requires careful sampling and advanced statistical modeling to assess temporal dynamics as well as detailed information on disease severity.

We have employed multiplex bead-based arrays to measure circulating serum levels of 74 unique analytes and explored the data from different angles. Firstly, data collected at the first available time point were analysed against overall outcome (moderate/severe/fatal) to classify early inflammatory status and assess their capacity to predict ensuing disease severity. Secondly, longitudinal data were analysed between outcome groups as well as against actual daily severity to identify analytes associated with severe disease. Thirdly, analytes significantly associated with severity during continued active disease were compared to those associated with disease severity during clinical improvement. Finally, samples collected at discharge and during convalescence were compared to healthy controls.

## Materials and methods

### Cohort study

The BEAT-COVID cohort was collected at the LUMC, Leiden, The Netherlands. Individuals diagnosed with PCR-positive COVID-19, who required hospital admission between April 2020 and March 2021 were invited to participate^17–20^. The majority of infections was with Wuhan-like viruses; the Netherlands experienced < 1% circulating alpha variant until January 2021 nationally (in Dutch: https://www.rivm.nl/corona/actueel/virusvarianten).

Clinical care was provided according to local and national guidelines and not influenced by study participation. Initially (April-Aug 2020), care was supportive only (wave-1, March 2020–August 2020), however, from August 2020 onwards patients received dexamethasone (wave-2, August 2020–March 2021). Since our study was initiated shortly after the onset of the pandemic in The Netherlands, criteria for hospital admission were also different between the waves. In wave-1 hospital admission was limited to the more severe cases, and generally admission occurred later after onset of symptoms compared to wave-2 as a result of capacity and national guidelines. In addition, SARS-CoV2 PCR testing was only available for hospital admitted patients whereas it was mandatory for all individuals with symptoms in the 2nd wave; hence, people were aware of the infection earlier and may thus have behaved differently.

Patients were included both on the regular ward as well as on the ICU. Inclusion criteria were: admission at LUMC, SARS-CoV-2 PCR positive, and minimum 18 years old. Exclusion criteria were: no informed consent from the patient or a representative. All participants were unvaccinated against SARS-CoV-2. Upon signing informed consent, blood samples were collected 3 times (Monday–Wednesday–Friday) a week during hospitalization and at an outpatient visit 6–12 weeks post discharge. Statistical sample size calculation was not performed, the sample size was determined based on availability. Anti-IL6R antibodies were only used in a few patients during our study.

In addition to the patients with COVID-19 disease, a control group of healthy individuals was collected in July 2020 for comparative analyses. None of these healthy volunteers had experienced the infection yet, as was confirmed by serological analyses Semi-quantitative detection of SARS-CoV-2 anti-nucleocapsid (N) protein IgG antibodies was performed on the Abbott Architect platform^21,22^. In this antibody chemiluminescent microparticle immunoassay (CMIA) test, the SARS-CoV-2 antigen coated paramagnetic microparticles bind to the IgG, respectively, IgM antibodies that attach to the viral nucleocapsid protein in human serum samples. The Sample/Calibrator index values of chemiluminescence in relative light units (RLU) of 1.40 (IgG assay) respectively 1.00 (IgM assay) and above were considered as positive per the manufacturer’s instructions. Samples were collected from 8 males and 4 females (as the ratio of hospital admissions at that time was 2:1), with a median age of 64 (range 60–72). Healthy volunteers were sampled 5 times, also at 2 day intervals and samples were processed identically to those from the individuals included in the BEAT-COVID cohort.

Ethical approval for the study protocol was provided by LUMC Medical Ethics committee (protocol NL73740.058.20). The study was registered at the International Clinical Trials Registry Platform no. NL8589 (https://trialsearch.who.int/Trial2.aspx?TrialID=NL8589). The study complied with the latest version of the Declaration of Helsinki. The principal investigator had access to information to identify individual patients.

### SCODA-severity-scores

The Severity of COronavirus Disease Assessment (SCODA) disease severity-score was developed to track day-to-day disease severity in hospital admitted COVID-19 patients^23^. The score was based on the 4C mortality score, but constant parameters were substituted with parameters associated with breathing and oxygenation to better reflect the daily condition and its changes^23^. The SCODA score was developed such that there was continuous scoring during admission on the ward and ICU to permit longitudinal assessment of severity during both types of admission. The SCODA score consisted of the following parameters: respiratory rate, peripheral oxygen saturation on room air (ward only), P/F Ratio (ICU only), oxygen flow (ward only), FiO2 (ICU only), Glasgow Coma Scale (GCS), blood urea level and C-reactive protein (CRP)^23^. The most severely ill patients admitted to the ICU could reach a maximum severity-score of 17.

We defined the recovery time point, per definition after the highest severity-score for that individual patient, as a SCODA score of a daily severity-score of ≤ 7, with no subsequent increases. SCODA scores were included in the analysis when matched serum samples and measurements on the soluble analytes were available.

### Serum samples

Blood samples were collected by venipuncture including a 8.5 ml SST Vacutainer tube (Becton Dickinson, VWR, Amsterdam, The Netherlands). Upon clotting samples were centrifuged (10 min, 3000 rpm) within one hour by the central clinical chemistry laboratory and aliquoted for research purposes. Samples were stored at − 80 °C to guarantee optimal sample quality.

### Multiplex bead array

The following cytokine and chemokine reagent kits were selected to analyse the sera of the study cohort; the Bio-Plex Pro human Chemokine panel (40-plex, #171AK99MR2), Bio-Plex Pro human Cytokine Screening panel (48-plex, #12,007,283), Bio-Plex Pro Human Inflammation panel (37-plex, #171AL001M) and a custom made panel of IL-17F, IL-21, IL-23, IL-25, IL-31, IL-33 of the Bio-Plex Pro human Th17 cytokine panel (all Bio-Rad, Veenendaal, the Netherlands). Assays were performed according to manufacturer’s instructions with manufacturers specific standards and QC control samples. All samples were thawed and diluted 1:4 in sample Diluent HB and run as single measurement with the streptavidin PE (1:200, Becton Dickinson, Erembodegem, Belgium) detection label. Samples were acquired on a Bio-Plex 200 system and analysed with Bio-Plex manager software v6.2. In total 131 analytes were measured, in case measurements were out of range or zero for more than 75% of the samples, analytes were removed from the analysis. When analytes were present in more than one assay panel, the analyte with the largest dynamic working range was selected for downstream analysis to ensure maximum detection window, this resulted in data analysis on 74 unique markers.

Samples were coded, stored directly at − 80 °C upon collection and not thawed before the first measurement, to minimize the loss of detection due to low level or instable analytes. Measurements were performed continuously when 76 samples were available from the study. Thus, longitudinal samples from an individual were not necessarily measured at the same time but throughout the study period. To ensure technical or inter-assay variation would not impact the analysis, a reference control sample, generated by pooling sera of 4 severely ill COVID-19 patients was included in all assays. The reference control was aliquoted to avoid repeated freezing and thawing cycles, maintaining the quality of the reference sample. After each acquisition all standard curves were checked for performance, outliers were deleted and curve fitting optimized (Regression Type: Logistic—5PL with standard recovery between 70 and 130%). Datapoints out of range below the LLOQ were set to 0 pg/ml and above the ULOQ were set to maximum (200.000 pg/ml).

Evaluation of the reference control sample showed limited variation in the calculated concentrations for most analytes measured between assays (analytes: n = 74), assays: 11 plates; median %CV = 56.7% (Supplementary Fig. 6A). Analytes with large %CV were mostly produced in high levels with standard curve characteristics of a steep slope and less sensitivity in the lower ranges, impacting the calculated concentration even with minimal fluorescence differences.

### Data analysis and modelling

#### Analysis at first time point

Normalized soluble marker levels were + 1 (to avoid zero as possible outcome), log2-transformed. Data was visualised and modelled using R (R Core Team, 2023), RStudio (Posit Team, 2023) and packages (R Core Team, 2023)*, ggplot2* (Wickham H, 2016)*, ggdendro* (De Vries A, Ripley BD, 2022)*, mixOmics* (Rohart F, Gautier B, Singh A, Le Cao K-A, 2017)*, caret* (Kuhn M, 2008)*, glmnet* (Friedman J, Tibshirani R, Hastie T, 2010)*, psych* (Revelle W, 2023)*, pROC* (Robin X, Turck N et al., 2011)*, ggpubr* (Kassambara A, 2023) and *GLMMadaptive* (Rizopoulos D, 2023)*.* Statistical testing comparing medians of two groups was performed using Mann–Whitney U. In addition, for analysis of differential soluble analyte levels between disease outcome groups and healthy controls or between waves Mann–Whitney U was performed and p values were adjusted for multiple testing by Benjamini–Hochberg correction. The heatmap is clustered row-wise based on complete-linkage hierarchical clustering. Supervised dimensionality reduction was carried out using partial least squares-discriminant analysis (PLS-DA), separating disease outcome groups and correlation analyses were performed using Spearman’s Rank and Benjamini–Hochberg correction was performed on p-values.

#### Machine learning analysis

For identification of the best-classifying soluble analyte signatures-profiles to model outcome (fatal outcome yes or no), ICU admission, high maximum disease severity and time to disease recovery at the earliest available time point (closest to hospital admission), logistic regression with lasso regularisation was performed. Leave-one-out cross-validation and train-test split (training set = 70%, test set = 30% of dataset) were used to assess the performance of the trained regression models. The classifying performance was further assessed by evaluating sensitivity, specificity, receiver operating characteristic (ROC) curve and area under the ROC curve (AUC).

#### Longitudinal data analysis

The progression over time of each soluble analyte was modelled using linear mixed effects models for the total cohort. Linear mixed models are an extension of the simple linear regression models when multiple measurements on the same subject are collected and use random effects to capture the serial correlation. Wave of sample collection was included as confounder into the model. To enhance comparability of the patients and stages of the disease process the number of days since disease onset was used for the timing of the samples within each patient. The non-linear progression over time was captured using natural cubic splines. To compare the progression between the different outcome groups the main effect and the interaction term between the days since disease onset and the outcome groups was added in the model. Similarly to explore association of each soluble analyte with the disease severity the main effect and the interaction term between the days since disease onset and disease severity was added in the model. For soluble analytes with values outside the limits of detection, the truncated normal distribution was used. Finally to capture the serial correlation random intercept and random slope terms were used.

Pathway analyses for exploratory purpose were performed in Ingenuity Pathway Analysis (Qiagen, Germany) with input of 40 significantly different analytes in a core analysis with the Ingenuity Knowledge database as reference set. The 40 significant analytes were selected based on the association between circulating levels and daily SCODA severity-score tested over time to identify different mean progressions during follow up.

## Results

### Cohort description

**Figure 1:**
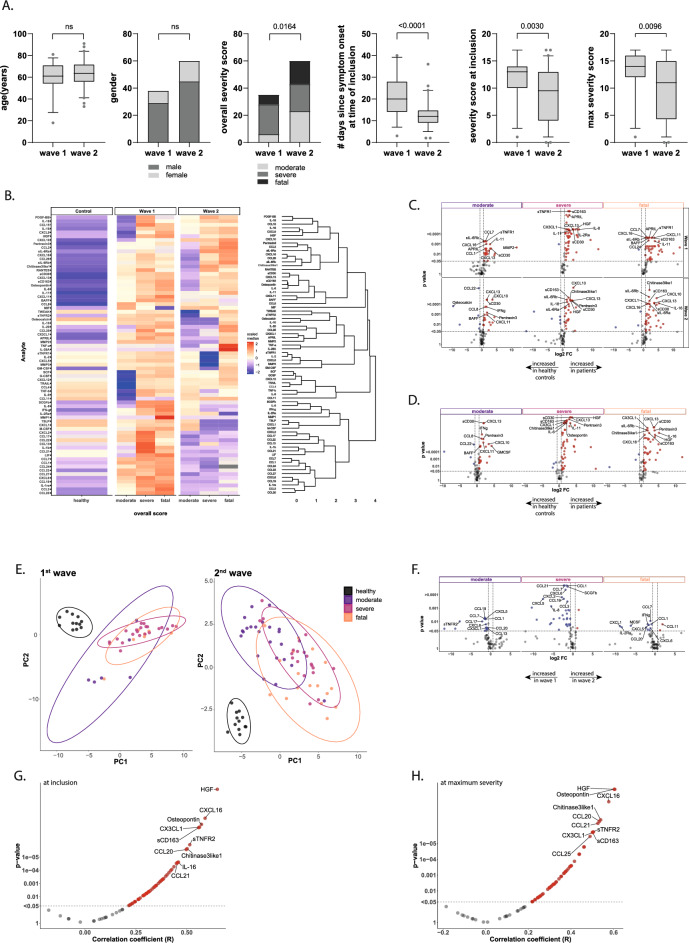
Serum analytes at hospital admission differ over outcome groups. **A** Descriptive characteristics of BEAT-COVID participants, 95 patients (and 12 healthy controls). Sample collection in 2 waves (1: April–August 2020; 2: August 2020–March 2021), participant characteristics are plotted for age, gender, outcome (moderate (hospital admission, no ICU), severe (ICU admission during hospitalisation) and fatal (patient deceased as result of SARS-CoV2 infection)), days since symptom onset at first time point of sample collection (= inclusion) and SCODA-severity-scores at first and maximal time points. Box and whiskers summarize the median (thick line), 25–75% percentiles (box) and 5–95% percentiles (whiskers), outliers are shown. Significant differences between groups were identified using Mann–Whitney-U-tests and for gender and overall severity score by Chi-square testing. **B** Heatmap of relative levels (medians) of all circulating analytes, data collected in pg/ml, + 1log2-transformed and row-scaled. Data are separated for wave-1 and 2, and outcome groups in separate columns. Complete-linkage Euclidian hierarchical clustering was performed with resulting dendograms. **C** Volcano plots of differences in analyte levels for moderate, severe and fatal outcome groups relative to the healthy control group, for wave-1 (top) and wave-2 (bottom). Difference was calculated using Mann–Whitney-U-test, with Benjamini-Hochberg (FDR), significant markers in red and blue. The top 10 markers were named. **D** Volcano plot of differences in combined data collected in wave-1 and 2. Analysis and plotting same as 1C. **E** PLS-DA analysis of healthy controls and 3 outcome groups (moderate, severe and fatal) for wave-1 (left) and wave-2 (right). **F** Volcano plots of differences in analyte levels for moderate, severe and fatal outcome groups in wave-1 vs wave-2. Analysis and plotting same as 1C, **G** Correlation analysis (Spearman) of analyte levels and disease severity at the first available time point for each patient with P-values on the y-axis against the correlation-coefficient on the x-axis. Significant analytes (*P* < 0.05, correlation-coefficient > 0.2) in red and top 10 markers named. Severity-scores were calculated on daily basis, per individual patient the time point with the highest daily severity-score was selected (if multiple days with a similar high score the first day was taken) and correlation analysis (Spearman) and plotting was performed as in 1G.

The BEAT-COVID cohort investigated here included the first 95 individuals with serum samples collected longitudinally during hospital admission and following discharge (total cohort size 191 individuals). Longitudinal sampling resulted in 252 samples from 35 individuals in wave 1 and 284 samples from 60 individuals in wave 2. Individuals had a median age of 63 years (61 years (wave-1) and 63.5 years (wave-2)) (Fig. 1A). Our cohort included more male than female participants reflecting the hospital admitted population (74 males, 21 females full cohort; 29 males, 6 females in wave-1; 45 males, 15 females in wave-2) (Fig. 1A) (all data provided in supplementary file, raw data). In addition to the daily severity-scores which are used for longitudinal analyses, individuals were categorized based on their overall disease course and outcome into moderate, severe and fatal disease. Moderate disease was defined as hospital admitted with PCR proven SARS-CoV2 infection on the regular ward, never requiring admission to the ICU and hospital discharge. Severe disease was defined as COVID-19 disease requiring ICU admission in some part of treatment but with discharge as outcome. Finally, fatal outcome was defined as all individuals with hospital admission due to SARS-CoV2 who succumbed to the disease. Individuals in wave-1 comprised 6 patients with moderate disease, 22 with severe disease and 7 patients with fatal outcome and in wave-2 23 moderate, 20 severe and 17 fatal disease patients (Fig. 1A). The number of days since onset of symptoms was higher in wave-1 (median 20 days) compared to wave-2 (median 12 days) (Fig. 1A). At the time of hospital admission, patients in wave-1 had higher severity-scores (median 13 for wave-1 vs 9.5 for wave-2) (Fig. 1A). Moreover, the maximum daily severity-score reached was also higher in wave-1 (median severity 14) compared to wave-2 (median severity 11) (Fig. 1A).

### Circulating analyte levels associate with disease severity

Soluble markers were measured in serum (in pg/ml) and concentrations extrapolated using standard curves^24^. All data were considered to be non-normally distributed as not all analytes reached normality (supplementary file raw data). First, we assessed the levels of soluble markers in the first available sample after hospital admission in comparison with age- and sex-matched healthy controls. A heatmap with hierarchical clustering describes the levels of soluble analytes for the disease outcome groups (overall outcome), separately for wave-1 and 2 (Fig. 1B). In the first wave, individuals with moderate disease differed from healthy, uninfected controls, by increased levels of proinflammatory chemokines including CCL2, CCL7, CXCL16, RANTES, IL-6, CCL8 and markers of T-cell and macrophage activation such as sCD30 and sCD163, as well as decreased levels of markers such as G-CSF, TRAIL, CXCL12 and TNF-β. Univariate plots for several analytes are shown in supplementary figure S1. In severe and fatal disease increased serum levels of IL-18, CCL15, CCL9 were detected and strong increases in levels of IL-1β, CCL13 and CCL21 were observed, without a decrease in G-CSF, TRAIL, CXCL12 and TNF-β (Fig. 1B). In wave-2, overall soluble marker levels were less increased compared to the healthy controls, in particular individuals with moderate disease differed less from the healthy, uninfected population. Patients with severe and fatal outcomes also showed less perturbed inflammatory marker profiles (Fig. 1B). IL-28A and TNF-α levels were highest in individuals with fatal outcome.

Comparative analysis of serum marker levels was performed, at the first available time point, relative to the healthy control population and separated for outcome and wave. In wave-1 significance was reached for 28 analytes in moderate, 54 in severe and 43 in fatal disease. In wave-2, serum levels of patients with moderate disease differed in 25 analytes, severe patients in 33 and in fatal patients in 37 analytes from healthy controls (Fig. 1C). Combination of both waves resulted in identification of 27 analytes, at the earliest available time point, that were significantly different in individuals with moderate outcome, 50 in severe and 45 in individuals with fatal outcome compared to healthy controls (Fig. 1D).

As waves 1 and 2 were intrinsically different in particular regarding treatment strategies we decided not to correct for treatment as covariate factor for the primary analysis. However, additional confounding factors could have contributed to analyte expression patterns. Therefore, we have modelled analyte levels at time of admission, in the total cohort, for five covariates (BMI, diabetes status, antibiotics, antivirals, corticosteroids) using linear regression and ANOVA with Benjamini–Hochberg correction of the p values. Administration of antivirals and especially corticosteroids contributed significantly to the levels of 44 analytes, most of them were also significantly different between wave 1 and 2, assuring that those differences were the result of the treatment. Analyte levels were not affected by BMI and a diagnosis of Type 2 Diabetes, whereas use of antibiotics only influenced osteopontin levels (supplementary file analysis).

PLS-DA analysis at the earliest available time point in wave-1 as well as wave-2 discriminated all disease outcome groups from the healthy control population. However, the disease outcome groups overlap largely (Fig. 1E). Since wave-1 and 2 had different characteristics, including duration of symptoms and disease severity but also corticosteroid treatment, we performed a direct comparison of circulating analyte levels between the waves. Patients with moderate and severe outcomes had significantly increased soluble analyte levels in wave-1 compared to wave-2, with the most abundant differences in individuals with severe disease (Fig. 1F). In patients with fatal outcome there was only a small number of analytes significant between wave-1 and 2.

The SCODA daily severity-score correlated for 57 analytes with serum levels (Fig. 1G). At the time point with the highest individual severity-score, a total of 53 soluble analytes correlated with disease severity (Fig. 1H). Boxplots for a selection of markers were plotted (supplementary Fig. 2).

### Circulating analytes at hospital admission have predictive value for disease severity

**Figure 2:**
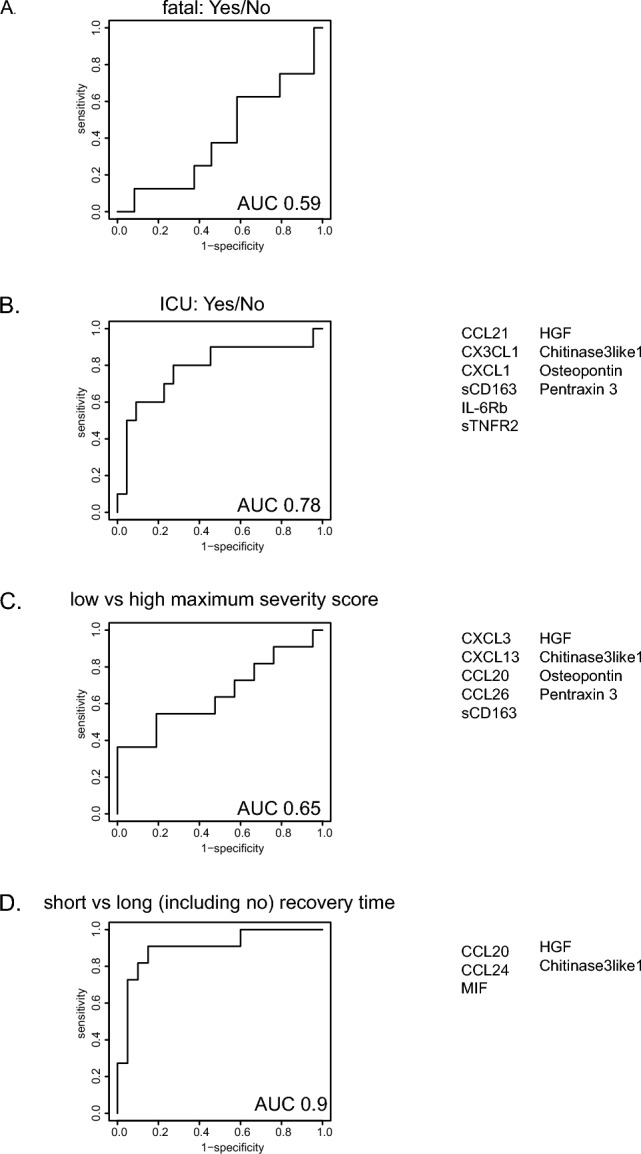
Prediction of disease course at time of study inclusion. ROC curves showing the predictive power of the analyte signatures identified to predict fatal outcome (A), ICU admission (B), severity-score (C) and disease duration (D), using logistic regression with lasso regularisation and LOOCV and train (70%)-test (30%) split to assess performance. For severity-scores the cut-off was set at a value of 8 and for disease duration day 21 was used. ROC curves were generated separately for all data of both waves combined to reach sufficient power. Individuals included in generation of the predictive algorithms varied. Fatal outcome yes (n = 8) vs no (n = 24), ICU yes (n = 22) vs no (n = 10), low (n = 11) vs high (n = 21) maximal severity-score, short (n = 11) vs long (n = 20) recovery time. AUC are included in each of the ROC curves and markers included in the signatures are described next to the ROC curves for all predictions with an AUC > 0.6.

We employed machine learning on the soluble analyte data collected at the earliest available time point (closest to hospital admission) to predict fatal outcome, ICU admission, high maximum disease severity and long time to disease recovery. Soluble marker signatures at the early time points were not able to predict outcome as defined by fatal outcome or survival (Fig. 2A). The need for ICU admission during hospitalisation was predicted by the soluble analyte profile at the first available time point, reaching an AUC of 0.78 (Fig. 2B). A high daily SCODA disease severity-score within each individual was predicted with an AUC of 0.65; since we set the cut-off at a maximum severity-score of 8, the group with low maximum severity was relatively small (n = 20 for training and n = 11 for testing) which is not ideal for the machine learning algorithm (Fig. 2C). Finally, we assessed whether a fast vs. slow/no recovery (before or after day 21), could be predicted at the earliest available time point after hospital admission. The algorithm was successful in prediction short vs. long or no recovery times at hospital admission, with an AUC of 0.9 (Fig. 2D). The large overlap between the predictive signatures identified is not surprising as the ‘states’ we aimed to predict are not unrelated, individuals with more severe disease are more likely to require ICU care and have longer recovery times.

### Longitudinal cytokine profiles

**Figure 3:**
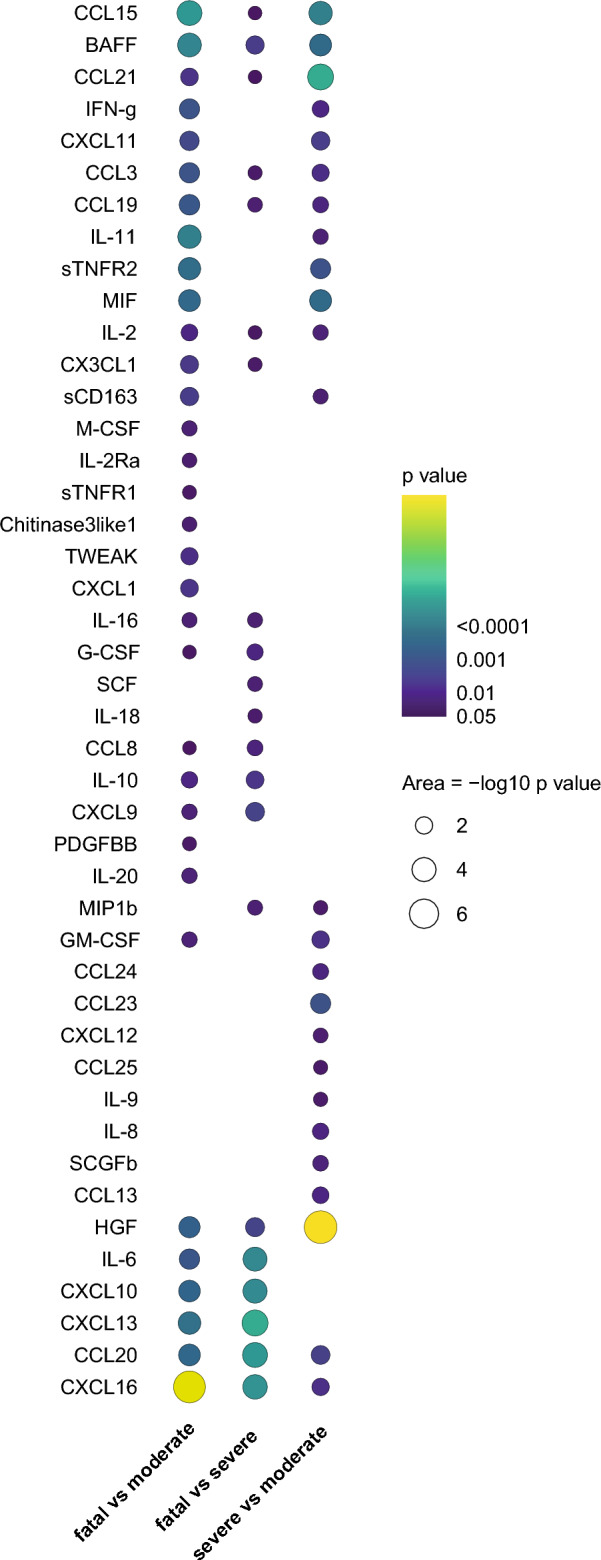
Longitudinal trajectories differ between disease outcome groups. Longitudinal modelling linear mixed effects models were applied on plus one log2-transformed values of each soluble analyte separately. For each analyte the association between circulating levels and outcome categories (moderate, severe, fatal) was tested to identify different mean progressions during follow-up. Pairwise comparisons were made between moderate and severe, severe and fatal and moderate and fatal outcome groups. *P*-values are plotted when significant per analyte in each comparison, with dot size and colouring reflecting *P*-value.

To obtain further insights into the relation between soluble analytes and disease severity, longitudinal models, including wave as covariate and time since symptom onset as factor, were employed. Longitudinal data were plotted for all individuals and grouped by ultimate outcome. Subsequently, mean profiles were fitted over time for each of the defined outcomes (moderate/severe/fatal). Finally, fitted mean profiles were compared and analysed for statistical differences in their slopes over time (examples in Supplementary Fig. 3). Significant differences in soluble analytes were identified between moderate and severe, severe and fatal as well as moderate and fatal outcome groups. As expected the most significant differences in longitudinal analyte expression were identified between moderate and fatal outcome (Fig. 3, supplementary file analysis). The highest significance was observed for CXCL16, CXCL13, CXCL10, CCL20, HGF, CCL15 and IL-6, similar to markers associated with severity at hospital admission. Interestingly, 60 analytes showed different profiles over time and most analytes had statistically different slopes between all groups compared (Fig. 3).

**Figure 4:**
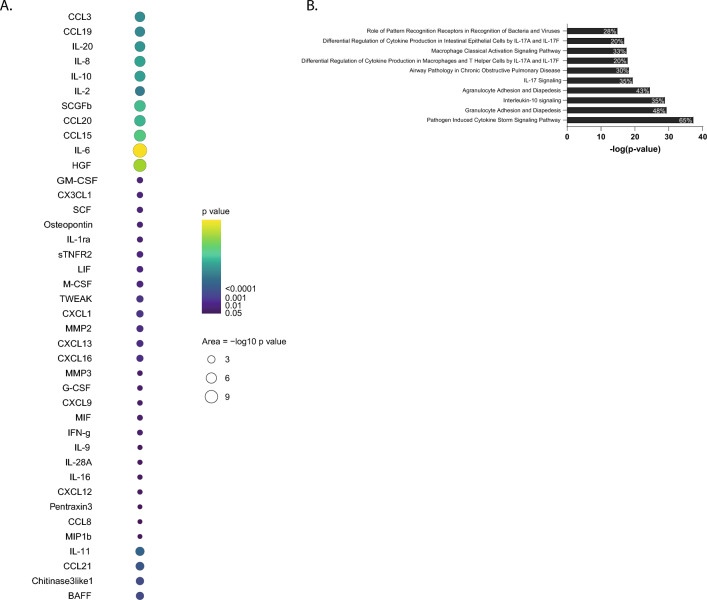
Correlation of analyte levels with disease severity. **A** Longitudinal modelling linear mixed effects models were applied on plus one log2-transformed values of each soluble analyte separately. For each analyte the association between circulating levels and daily SCODA severity-score was tested over time to identify different mean progressions during follow up. P-values are plotted when significant per analyte, with dot size and colouring reflecting *P*-value. **B** Pathway analysis was performed in the Ingenuity platform on all analytes with a significant association between severity-scores and analyte levels longitudinally (from A) and pathways were identified that contained these analytes. The top 10 canonical pathways, based on P-values, are listed. In each bar the percentage indicates the proportion of the 40 analytes that correlated significantly and was associated with this pathway, thus 26 of 40 (= 65%) analytes are part of the ‘pathogen induced cytokine storm signaling pathway’.

To assess longitudinal patterns and thereby associations with severity dynamics during different disease stages we used linear-mixed models to assess the association of analyte levels with daily severity-scores over time, with wave as cofactor (Supplementary Fig. 4). Briefly, analyte progressions over time were plotted for the sample quantiles of SCODA severity-scores. Combined longitudinal assessment of the association between disease severity and analyte levels identified IL-6 as the strongest factor associated with severity, in total 40 analytes were significantly associated with severity-score over time (Fig. 4A). They were explored further using Ingenuity pathway analysis to identify canonical pathways when combining these analytes. High significance was found for ‘pathogen induced cytokine storm signaling’, ‘airway pathology in chronic obstructive pulmonary disease’ ‘IL-10 signaling' and multiple pathways related to ‘IL-17 signaling’ and ‘macrophage classical activation signaling pathway’ (Fig. 4B). Twenty-six of the 40 significant analytes (65%) were part of the pathogen induced cytokine storm pathway, indicating that this is very likely a major path in COVID-19. In addition, most other pathways also point towards strong inflammation, with likely involvement of macrophages.

### Analytes associated with severity differ in deteriorating versus recovery phase

**Figure 5:**
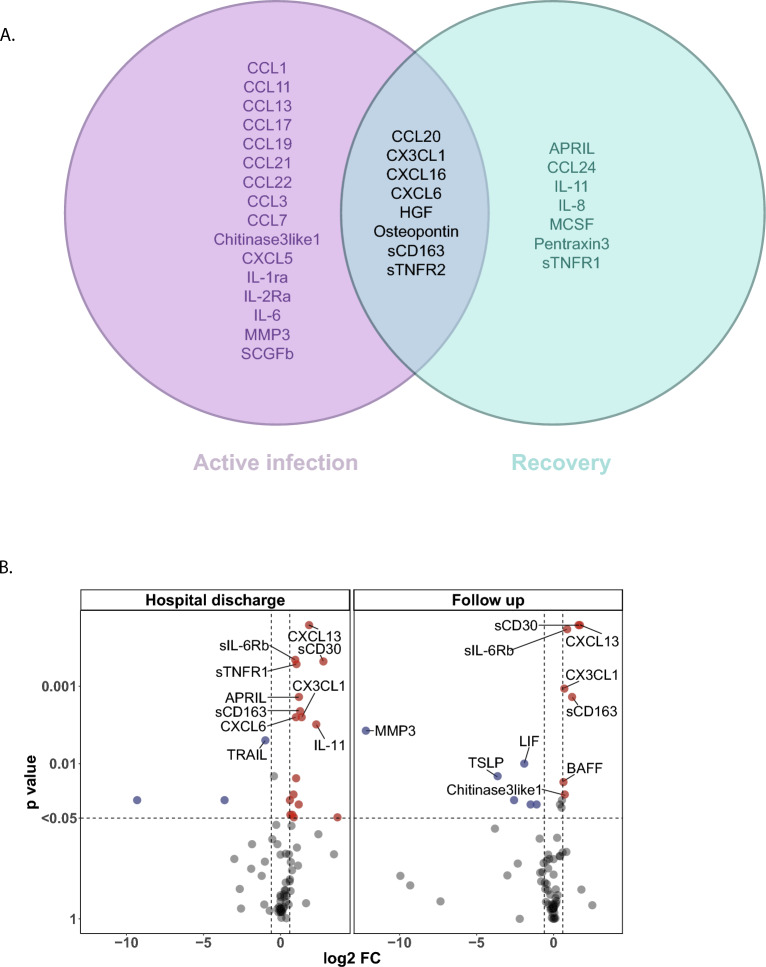
Different markers associate with disease progression and recovery. **A** Patients were hospitalized with progressing disease, however before hospital discharge recovery was initiated. The SCODA daily severity-score allowed to dissect between progressing/ongoing disease (increasing or stable daily severity-scores) and recovery (decreasing daily severity-scores). The time point of recovery was defined as a daily severity-score of ≤ 7, with no subsequent increases. Correlation analysis (Spearman, *P* < 0.05) was performed on analyte levels and daily severity-scores on all time points collected before recovery (progressing/active disease) and compared to analytes correlating at all time points post recovery, but still during hospital admission. Before recovery 57 samples were analysed, after recovery 34 samples were analysed. Data are displayed as Venn diagrams with analytes uniquely correlation in active infection, analytes overlapping between active infection and recovery, and analytes uniquely correlating with severity during recovery. **B** Samples collected at hospital discharge as well as samples collected on an out-patient follow-up time (6–12 weeks post discharge) were compared to healthy controls. Volcano plots of differences in analyte levels for discharge and follow-up relative to the healthy control group. − log10-transformed P-values are plotted on the y-axis against log2 FC on the x-axis, analytes with *P* < 0.05 and log2 FC < − 0.6 or > 0.6 were labelled as significant (red and blue dots). Difference was calculated using Mann–Whitney U, with Benjamini–Hochberg as FDR method. The top 10 analytes were named.

Correlations so far have been made over the entire time span of hospital admission. However, to understand the pathophysiology of disease it is interesting to separate analytes potentially involved in disease progression from those potentially associated with recovery. The SCODA disease severity-score also describes the ‘recovery time point’, the time point since symptom onset and after the highest individual severity-score that has a score of 7 or lower. We employed this to divide the samples in ‘active infection’ (all since hospital admission and before reaching the recovery point) and ‘recovery’ (all after the recovery time point, but still during hospital admission). Correlations were observed both in the active infection phase and the recovery phase (Fig. 5A). During the active infection phase 24 analytes showed a correlation with disease severity, while during recovery (but still hospital-admitted) levels of 15 analytes correlated with disease severity-scores.

The BEAT-COVID cohort also collected samples at 6 or 12 week follow-up visits post discharge. Samples at discharge (the last available time point during hospital admission) as well as at the follow-up time point were compared to healthy controls. At hospital discharge still 21 analytes were significantly different from the healthy control population (Fig. 5B, supplementary data files). Inflammatory markers such as CXCL13, sIL-6Rβ, sCD30, sTNFR1 and sCD163 were still increased. Intriguingly, 16 analytes were still increased in serum of convalescent patients at their follow up outpatient visit 6 to 12 weeks later, suggesting persisting or slowly resolving inflammation (Fig. 5B, supplementary Fig. 5).

## Discussion

COVID-19 disease is characterized by strong inflammation. Individual responses to infection vary enormously, ranging from asymptomatic infection to fatal disease. Here, we embarked on extensive longitudinal measurement of soluble analytes in serum, collected in two waves, to obtain insights in the pathophysiology of the disease but also aiming to identify markers with predictive value at hospital admission, that could guide clinical decision making.

Circulating analyte levels were measured by multiplex bead arrays on all available samples and results analysed in several ways using advanced, longitudinal computational models. As continuous scaling was critical, all sample collection time points were related to the onset of symptoms, rather than to hospital admission as most others assessing inflammatory profiles have done^4^. Firstly, we analysed levels of circulating analytes at the first available time point since study inclusion and compared these to outcome in categories (moderate, severe and fatal). Secondly, we correlated analyte levels at inclusion to the severity at that time point, as calculated with the SCODA severity-score^23^. Thirdly, predictive models were identified at time of inclusion aiming to predict outcome (survival vs fatal), disease severity (ICU vs no-ICU admission; high vs low max SCODA disease score), and duration of disease (long or no vs short recovery time). Fourthly, longitudinal models were developed to assess differences in analyte levels over time in the different disease outcome groups. Fifthly, disease severity was also modelled as continuous parameter over time to correlate with soluble analyte levels. Sixthly, analytes correlating with disease progression were compared to those associated with convalescence. Finally, soluble analytes at hospital discharge and outpatient follow-up were correlated to levels of healthy controls.

Surprisingly and intriguingly, the majority of analytes identified in all analysis strategies were very similar and typically overlapping. Analyte levels were characteristically higher with increasing disease severity, but the type of markers associated with different COVID-19 stages was very similar. Key markers that were upregulated in serum from hospital admitted individuals with COVID-19 disease were HGF, CXCL13, sCD30, CXCL16, IL-6, sCD163, IL-11, CXCL10, IL-16, CX3CL1, sIL-6Rβ, Osteopontin and Chitinase3like1, all associated with inflammatory responses, including cytokine storms, TLR activation and macrophage activation. Markers related to B cell or T cell immunity were not in the top analytes that were differently detected. Pathway analysis of all markers associated with disease severity over time also identified ‘pathogen induced cytokine storm signaling pathway’ as top hit, IL-10 and IL-17 signaling as prominent hits as well as ‘macrophage classical activation signaling pathway’. Even during convalescence, both at hospital discharge and outpatient follow-up, those markers were significantly increased compared to the healthy control population, suggesting persisting inflammation. Advanced longitudinal models, both based on outcome defined groups and continuous models on severity-scores also identified major associations with macrophage activation syndrome.

Analytes identified at the earliest possible time point (study inclusion) were remarkably similar to those at the most severe disease state, indicating that ongoing inflammation is the major determinator of circulating analyte profiles. When comparing samples during disease progression and convalescence in association to severity-scores, more analytes were detected during disease progression compared to convalescence. However, the markers significantly correlating with disease severity during convalescence, are consistent with ongoing inflammation rather than resolution of inflammation or recovery. HGF and CXCL13 were also previously identified as markers of severe COVID-19 and good predictors of ICU admission^5^. In particular HGF has been described to dampen inflammation as counter-regulator of many pro-inflammatory cytokines and may even promote tissue repair^5^. In our data, HGF was also amongst the strongest correlates with severe disease at inclusion into the study, at maximum severity, longitudinally and during recovery but not at hospital discharge or during convalescence. Others have described increased HGF and KRT19 levels during convalescence and suggested they have potential value for prediction of lung impairment post-COVID-19^3^. The observed differences in HGF associations with disease may result from cohort differences e.g. in disease severity but also in the definitions used to group the patients as well as potentially in timing of sample collection.

Comparison of wave-1 and 2 in our cohort, highlighted the high proportion of dexamethasone usage in the 2^nd^ wave as the major difference between wave-1 and 2, whereas none of the patients in the 1^st^ wave received steroids early in disease. We observed strong differences in circulating pro-inflammatory markers between the waves, with more pro-inflammatory cytokines upregulated in wave-1. In severely-ill patients 33 analytes were significantly different between wave-1 and 2, likely reflecting differential dexamethasone treatment. Similarly, also in COVID-19 patients who received dexamethasone circulating levels of pro-inflammatory cytokines decreased 3–4 days after treatment start^25^. In our cohort IFN-γ and CXCL10 were significantly decreased in wave-2 compared to wave-1, possibly also as a result of the steroid treatment. Most other analytes were also less increased in wave-2 compared to wave-1, fully supporting these observations.

The limitations of our study involves most importantly the fact that only a single cohort of COVID-19 patients was analysed, without replication cohort. Although in some aspects the two waves could be considered two subcohorts from the larger cohort, with differences in duration of disease and routine treatment, which were also analysed independently. In addition, we have analysed our data with several different approaches (longitudinal vs cross-sectional, group classification vs continuous severity assessment) and identified the same marker signatures, which in a way could serve as within cohort validation. To the best of our knowledge, no datasets are publicly available with longitudinal data that would allow replication of our associations between circulating analytes and disease severity. Unfortunately, we were not able to include patients with similar clinical presentation but uninfected by SARS-CoV2. Albeit our primary goal was to assess analyte levels in relation to disease severity in COVID-19 patients for optimal treatment stratification, it would have been highly relevant to assess the same in patients with other causes of respiratory distress or other inflammatory conditions. Our cohort was heterogenous, in time since symptom onset, disease severity, treatment policies etc., but nevertheless we identified strong associations between circulating analytes and disease severity. Finally, our analysis was limited to patients that were hospital admitted and did not include less severe, or earlier stage patients with SARS-CoV2 infection, which would have been a valuable addition in particular for evaluation of the predictive signatures.

In summary, longitudinal measurement of circulating analytes combined with daily assessment of disease severity using a specifically developed score demonstrated that COVID-19 disease reveals strong pro-inflammatory profiles, most probably as a result of strong macrophage activation, with dominant roles of IL-6, IL-10 and IL-17 signaling cascades, which are associated with disease severity and outcome.

### Supplementary Information

Supplementary Information 1.

Supplementary Information 2.

Supplementary Figures.

## Data Availability

All data is made available as supplementary data files, raw data and analysis**.**
