# Supplementary material for: Longitudinal soluble marker profiles reveal strong association between cytokine storms resulting from macrophage activation and disease severity in COVID-19 disease
Source: Sci Rep. 2024 Jun 5;14:12882. doi: 10.1038/s41598-024-63586-8 (PMC11153563; doi:10.1038/s41598-024-63586-8)
Supplement: Supplementary file 3 — Supplementary Figures. [file 41598_2024_63586_MOESM3_ESM.docx]

**Supplementary Figure 1**


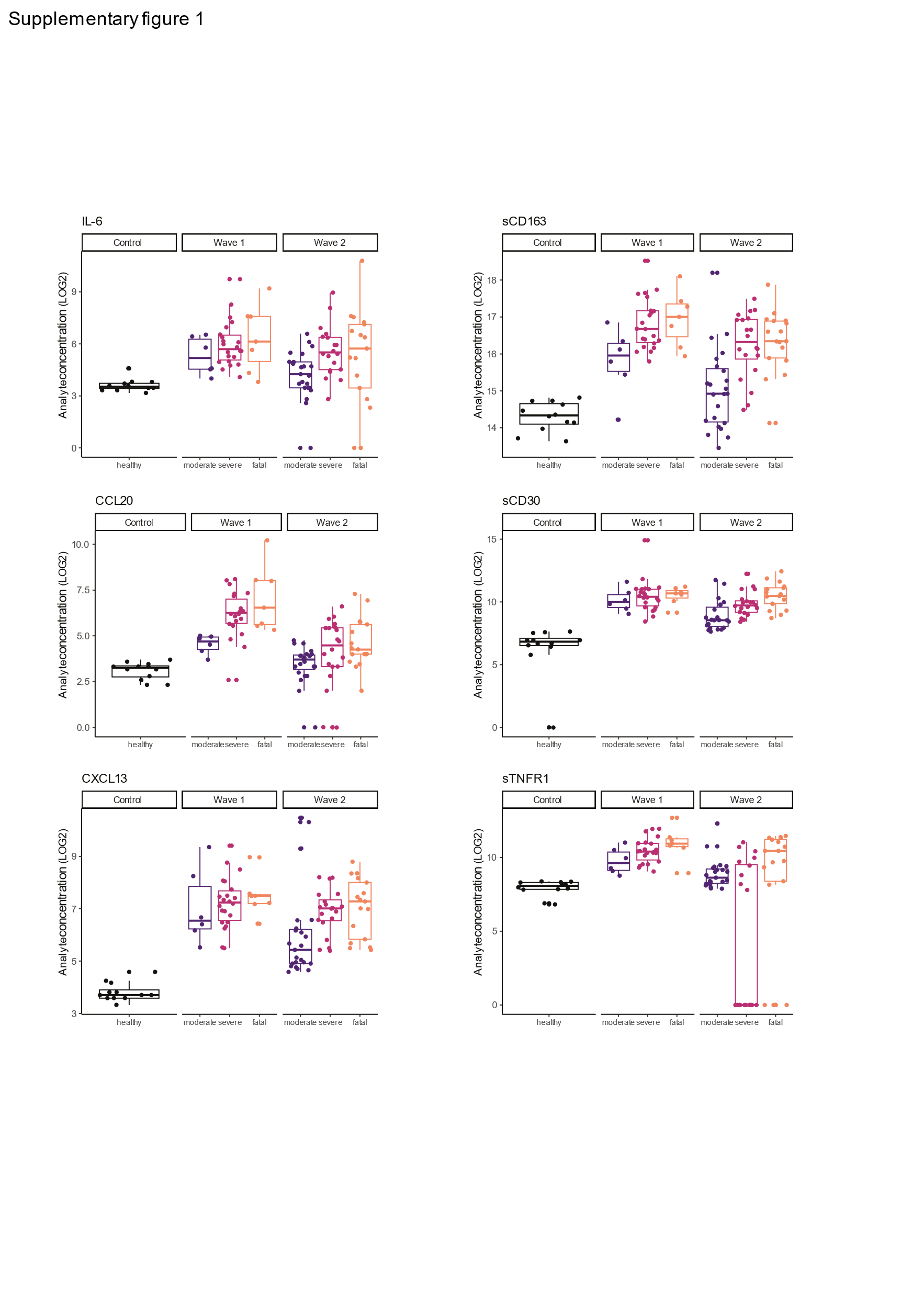
Univariate data of individual analytes in outcome groups over the waves at study inclusion

Analyte expression, measured at the first collected sample after hospital admission, is plotted for 6 individual analytes (IL-6, CCL20, CXCL13, sCD163, sCD30, sTNFR1) as examples for the healthy controls, moderate severe and fatal outcome groups separated for wave-1 and 2. Data are expressed as plus one log2 transformations of the concentration in pg/ml. All data points are plotted, boxes indicated the 25-75% percentiles with a line at the median and whiskers indicate 1.5*IQR.

**Supplementary Figure 2**


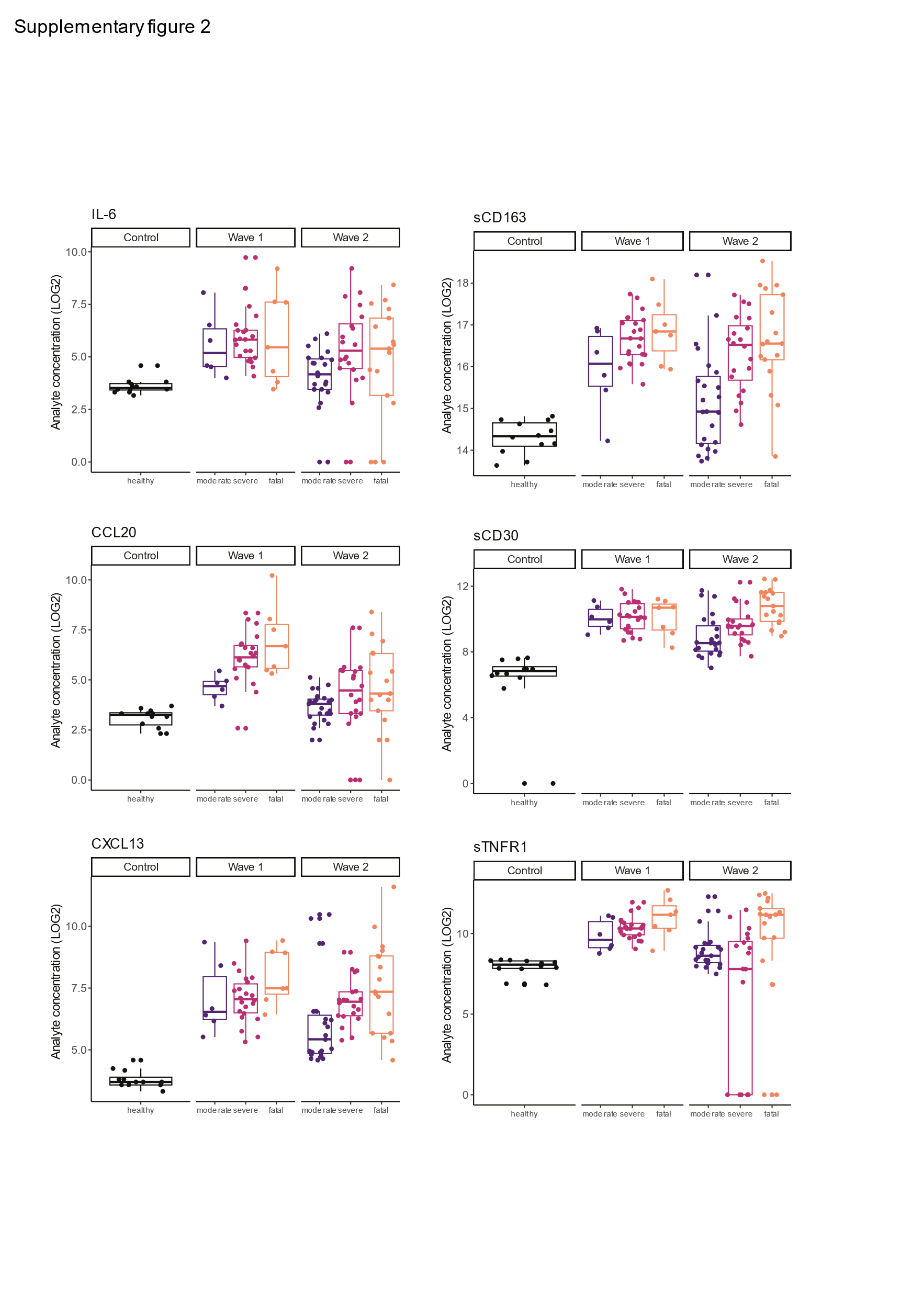


Univariate data of individual analytes in outcome groups over the waves at maximum severity

Analyte expression, measured at the time point the individual patient is experiencing maximum disease severity, is plotted for 6 individual analytes as examples for the healthy controls, moderate severe and fatal outcome groups separated for wave-1 and 2. Data are expressed as plus one log2 transformations of the concentration in pg/ml. All data points are plotted, boxes indicated the 25-75% percentiles with a line at the median and whiskers indicate 1.5*IQR.

**Supplementary Figure 3**


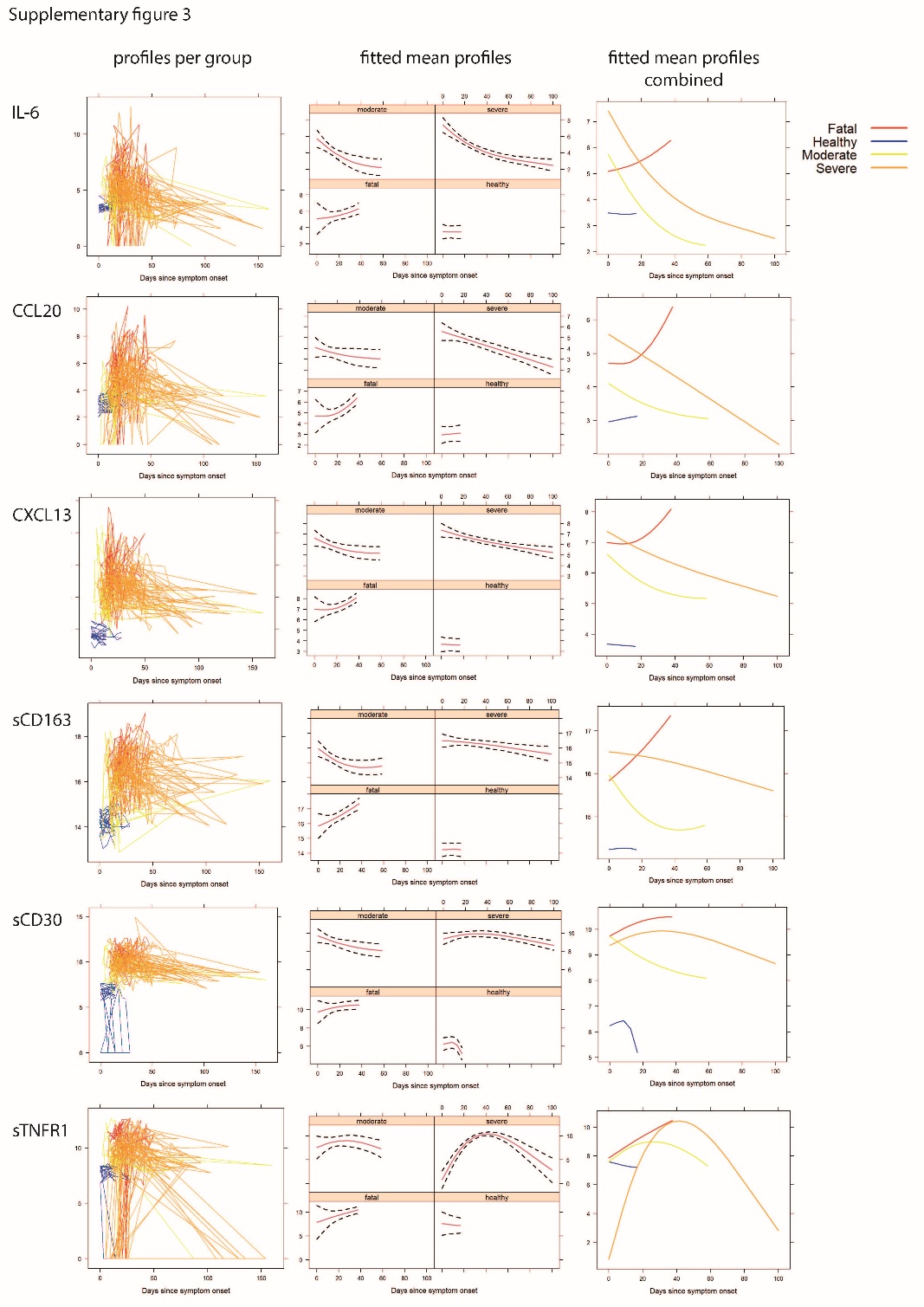


Longitudinal linear mixed effect models of soluble analytes

Analyte expression over time since onset of symptoms plotted for all individuals analysed (healthy individuals in blue, moderate outcome in yellow, severe outcome in orange and fatal outcome in red)(left panel). Profiles are fitted for mean analyte expression profiles within each outcome group over time, red lines indicate mean, black dashed lines indicate 95% pointwise confidence intervals for each group (middle panel). Combined graph of fitted mean profiles for the different outcome groups (right plots/panel).

**Supplementary Figure 4**


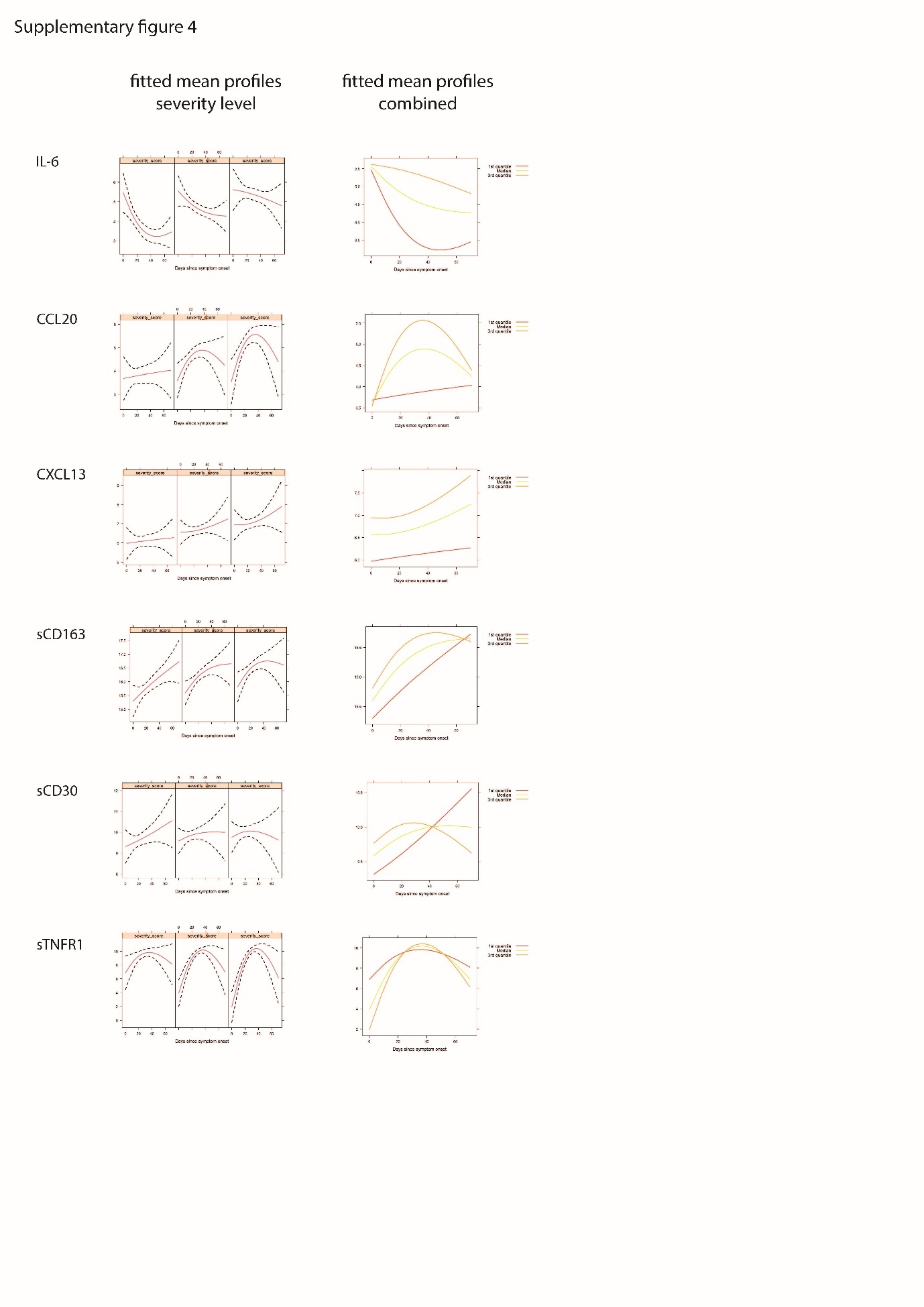


Longitudinal association between soluble analyte levels and daily disease severity (SCODA)

Analyte levels over time since onset of symptoms were fitted over the severity levels, red lines indicate mean, black dashed lines indicate 95% pointwise confidence intervals (left panel). Combined graph of fitted mean profiles for severity levels (right plots/panel).

**Supplementary Figure 5**


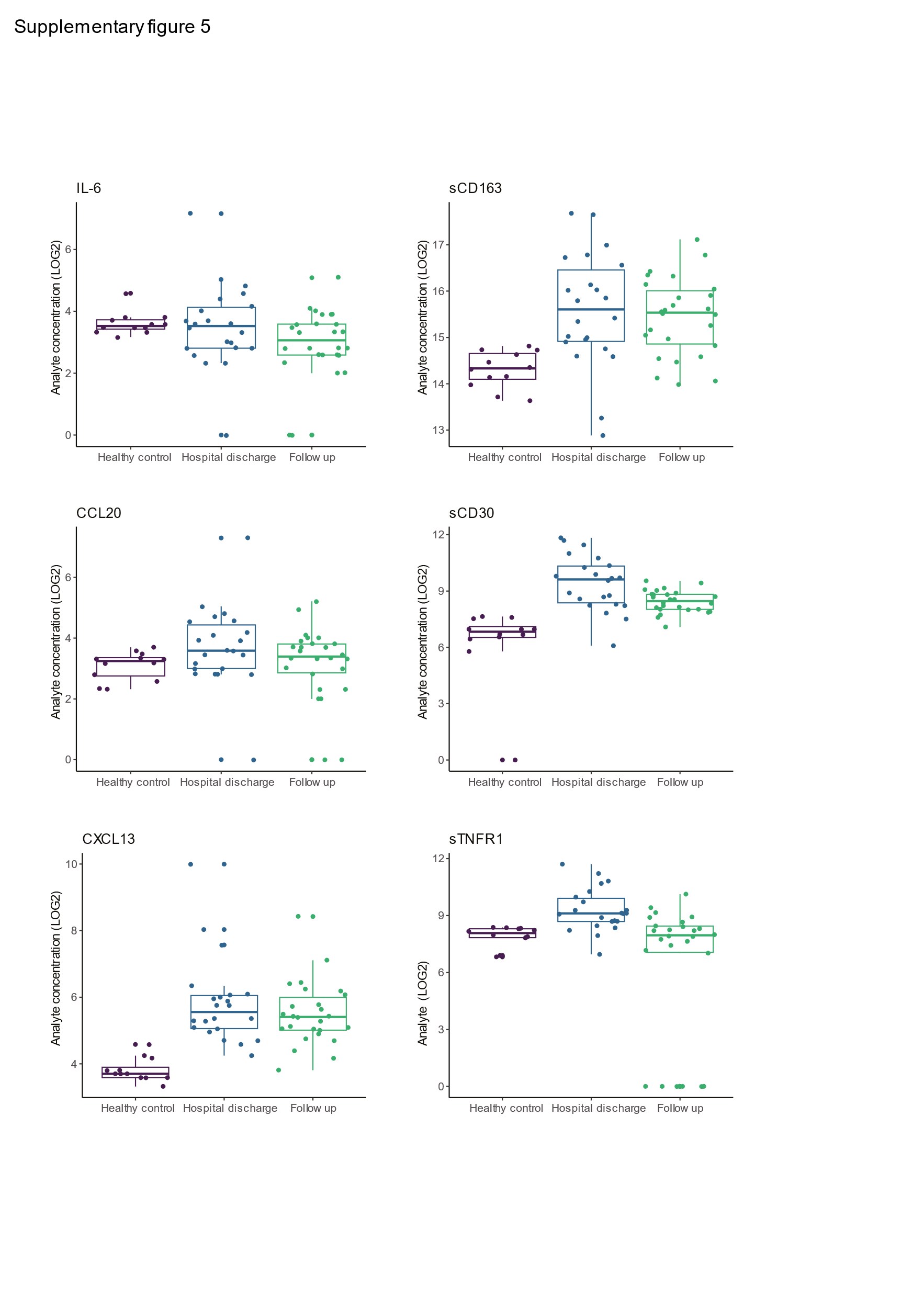
Soluble analytes still differ at discharge and follow up from healthy controls

Serum samples were selected at the last point of hospitalization and at a follow up outpatient visit 6-12 weeks later and circulating levels (log2 transformed) plotted relative to the healthy control population.

**Supplementary Figure 6**


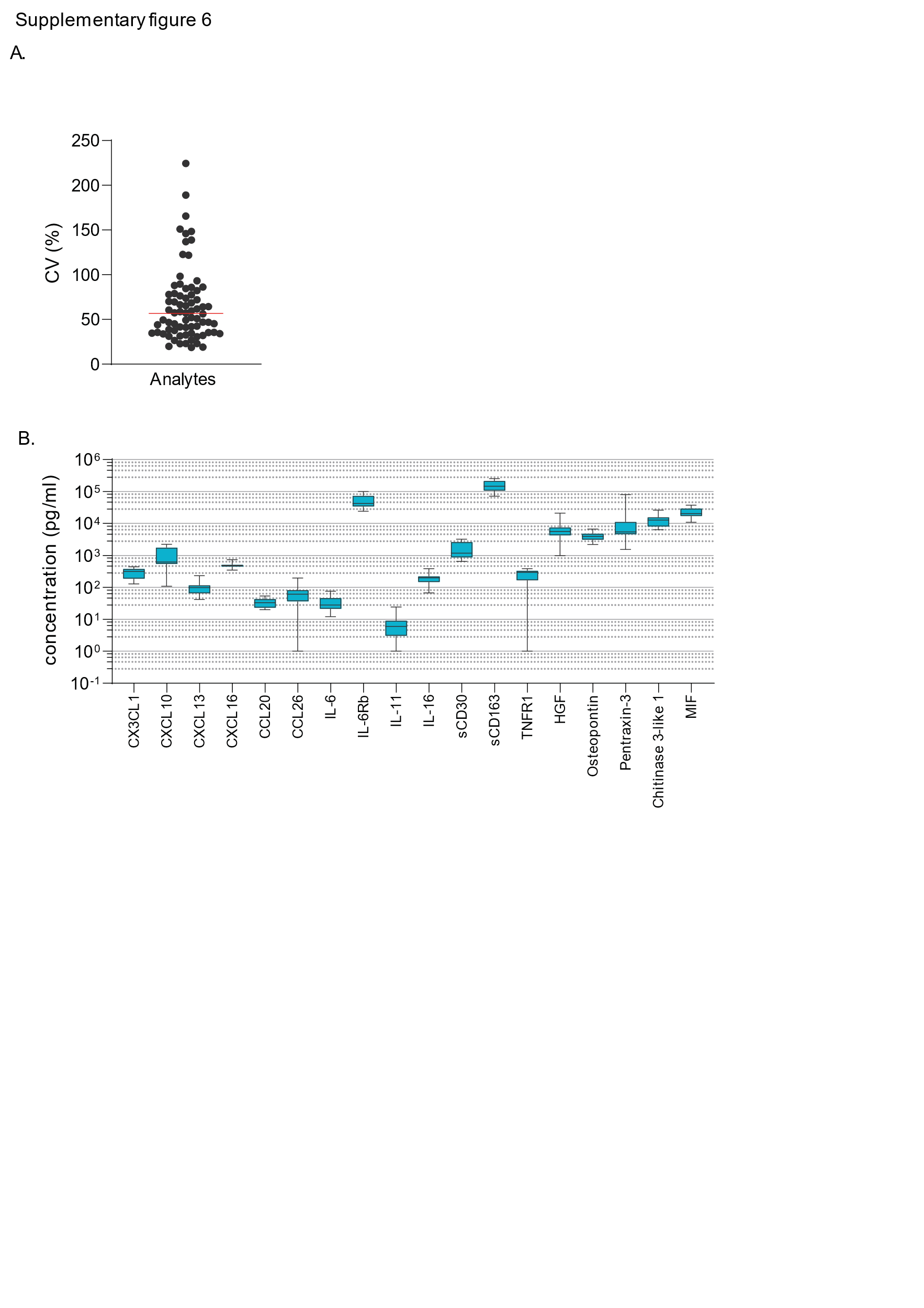
Reference control sample performance

An aliquot of the reference control sample was measured in each run for all analytes. In total 11 runs were performed and each analyte is represented by 11 data points.

1. Variation coefficients for the reference control dataset for all measured analytes were determined and plotted.
2. The reference control data for the key analytes (X-axis) is shown as boxes and whiskers, with boxes for the 5-95 percentile and whiskers for the minimum and maximum concentration (pg/ml) (Y-axis).
